# Systematical assessment of digit ratio in a female masculinization disease: polycystic ovary syndrome

**DOI:** 10.3389/fendo.2023.1146124

**Published:** 2023-05-08

**Authors:** Xueqi Yan, Aiqing Zhu, Yexing Li, Ziyi Yang, Yuteng Wang, Li Liu, Wei Liu, Dan Liu, Fenghua Li, Juan Du, Fang Cheng, Xueying Gao, Junli Zhao

**Affiliations:** ^1^ Center for Reproductive Medicine, Shandong University, Jinan, Shandong, China; ^2^ Key Laboratory of Reproductive Endocrinology of Ministry of Education, Shandong University, Jinan, Shandong, China; ^3^ Shandong Key Laboratory of Reproductive Medicine, Shandong Provincial Hospital Affiliated to Shandong First Medical University, Jinan, Shandong, China; ^4^ Shandong Provincial Clinical Research Center for Reproductive Health, Shandong University, Jinan, Shandong, China; ^5^ Shandong Technology Innovation Center for Reproductive Health, Shandong University, Jinan, Shandong, China; ^6^ National Research Center for Assisted Reproductive Technology and Reproductive Genetics, Shandong University, Jinan, Shandong, China; ^7^ Department of Reproductive Medicine, Yinchuan Maternal and Child Health Hospital, Yinchuan, Ning xia, China; ^8^ Department of Reproductive Surgery, Northwest Women’s and Children’s Hospital, Xi’an, Shanxi, China; ^9^ Reproductive Medicine Center, Department of Obstetrics and Gynecology, Tang Du Hospital, The Air Force Military Medical University, Xi’an, Shanxi, China; ^10^ Department of Reproductive Medicine, Yuhuangding Hospital Affiliated to Qingdao University, Yantai, Shandong, China; ^11^ Center for Reproductive Medicine, Ren Ji Hospital, School of Medicine, Shanghai Jiao Tong University, Shanghai, China; ^12^ Shanghai Key Laboratory for Assisted Reproduction and Reproductive Genetics, Shanghai, China; ^13^ Reproductive Medicine Center, General Hospital of Ningxia Medical University, Yinchuan, Ningxia, China; ^14^ Key Laboratory of Fertility Preservation and Maintenance of Ministry of Education, Ningxia Medical University, Yinchuan, Ningxia, China

**Keywords:** polycystic ovary syndrome, digit ratio, prenatal androgen exposure, hyperandrogenism, anatomical marker, sexual dimorphism

## Abstract

**Background:**

In recent years, the right ratio of 2nd and 4th digit length (2D:4D) is regarded as an anatomical marker of prenatal testosterone exposure. Polycystic ovary syndrome (PCOS) is a female masculinized disease and is determined by prenatal testosterone exposure. Whether the ratio in the right hand of PCOS women is reduced or not compared with non-PCOS women is under debate. To further investigate the relationship between PCOS and digit ratio, we systematically measured all the digit ratios.

**Methods:**

We recruited 34 non-PCOS women, 116 PCOS women, and 40 men and systematically measured all the ratios of digit length (2D:3D, 2D:4D, 2D:5D, 3D:4D, 3D:5D, and 4D:5D) of right hands and left hands.

**Results:**

Left 2D:3D, 2D:4D, and 2D:5D in men were significantly lower than those in non-PCOS women. Significantly lower digit ratios of left 2D:3D and 2D:4D were observed in PCOS compared with non-PCOS women. In the subgroup analysis, the left ratio of digit length in 2D:3D and 2D:5D of the hyperandrogenism subgroup was lower than that of the non-hyperandrogenism subgroup without statistical significance. The logistic regression model of PCOS revealed that 2D:3D, 2D:4D, 2D:5D, and 3D:4D of left hands were statistically related to the diagnosis of PCOS among all the digit ratios.

**Conclusion:**

Not only 2D:4D but also other digit ratios, such as 2D:3D and 2D:5D, are a marker of prenatal testosterone exposure and may be an anatomical marker of PCOS. The majority of these significant differences included left 2D, with the following order: non-PCOS women > PCOS women > men.

## Introduction

Digit ratio is an easily measurable anatomical marker of prenatal androgen exposure. The sexually dimorphic phenomenon of the relative ratio of the 2nd digit (2D, the index finger) and the 4th digit (4D, the ring finger) has been found since 1998 (1). That is, the length of 4D is longer in men and the length of 2D is longer in women and the digit ratio of 2D:4D tends to be lower in men. The ratio of 2D:4D is reported to be fixed early *in utero* and is negatively associated with prenatal testosterone (T) exposure (2, 3). Given the negative relationship between 2D:4D and prenatal T exposure, as well as the important influence of fetal T exposure on human disease, 2D:4D has been regarded as an anthropometric biomarker for abnormal prenatal T exposure in congenital adrenal hyperplasia (2, 4), prostate cancer (5, 6), and so on.

Polycystic ovary syndrome (PCOS) is a female masculinized disease and results from androgen exposure in the gestation stage. PCOS is characterized by oligo-ovulation/anovulation, clinical and/or biochemical hyperandrogenism, and polycystic ovaries detected by ultrasound, accompanied by acne and hirsutism (7). Animal studies revealed that androgen injection during pregnancy tended to have offspring presenting with the endocrine and metabolic phenotype of PCOS (8–10).

Hence, more studies focused on 2D:4D in women with PCOS (11–14), and the results were controversial. Some studies reported a subtle difference in the finger length pattern of women with PCOS, but a few studies found no difference for 2D:4D between PCOS and controls. Besides 2D:4D, other ratios of digit length were reported to present sexual differences in humans (15, 16). However, previous studies related to digit ratio and PCOS were confined to 2D:4D and lacked evidence of other digit ratios in PCOS. We hypothesized that 2D:4D is lower in PCOS and other digit ratios in PCOS are different from non-PCOS.

To test the hypothesis, we systematically investigated all the digit ratios of both left and right hands in non-PCOS women, PCOS women, and the men in this study. Hyperandrogenic (HA) PCOS and non-hyperandrogenic (NHA) PCOS subgroups were also analyzed to identify the potential effects of androgen on digit ratios.

## Materials and methods

### Sample

There were a total of 190 participants recruited from the reproductive center of General Hospital of Ningxia Medical University, namely, 34 non-PCOS women, 116 PCOS women, and 40 men. The diagnosis criterion was defined according to the 2003 Rotterdam criteria: oligo-ovulation/anovulation, clinical or biochemical hyperandrogenism, and polycystic ovary morphology diagnosed by ultrasound; two of the three items will be diagnosed. Diseases such as androgen-secreting tumors, Cushing’s syndrome, and congenital adrenal hyperplasia, which could cause hyperandrogenism, were the exclusion criteria. Non-PCOS women had normal hormone levels and regular menstrual cycle, who visited the IVF center due to male infertility or oviduct factors. Men from the population who visited the IVF center for normal physical examination or fertility requirements of their families were included; they have no relationship with non-PCOS or PCOS women. PCOS women were divided into a hyperandrogenism (HA) and a non-hyperandrogenism (NHA) group according to the testosterone concentration of 48.1 ng/dl as per the routine value of the hospital.

### Ethics statement

This protocol was approved by the Committee of General Hospital of Ningxia Medical University. All subjects had submitted a written informed consent.

### Digit ratio collection

The participants were asked to take photos of their right and left hands on the ventral surface using a Sony digital camera. With the help of ACDSee, digit lengths were marked in the line of digit axis: from a point in the proximal finger crease to the most distal point on the fingertip (17). The marked photos were printed and measured by vernier calipers from the photocopies. Given that the distance between camera and hands was not fixed, digit ratios rather than digit length could be included for further analysis. Eight digit lengths (the second, third, fourth, and fifth finger of the left and right hands) were measured at least twice to test the repeatability of the measurement. Based on these parameters, the following ratios of right and left hands were calculated: 2D:3D, 2D:4D, 2D:5D, 3D:4D, 3D:5D, and 4D:5D. The repeatability or intraclass correlation coefficient of digit length and digit ratio was high. The average value of measurements was used for subsequent analysis.

### Statistical analysis

Analysis on the differences in the digit ratio among non-PCOS women, PCOS women, and men was conducted. For normal distributed variables, one-way analysis of variance (ANOVA) and least significant difference (LSD) were applied to compare the continuous variables among these groups. The linear trends in the mean digit ratio were evaluated by calculating *p*-value for trend. For non-normal data, non-parametric Mann–Whitney *U* test and Kruskal–Wallis *H* test were used for comparison purposes. The *p*-values for difference and trend of digit ratio were adjusted by age. A logistic regression model was used to select the important variables with PCOS as the dependent variable and 12 digit ratios as the independent variable. All the digit ratios were input in the model with “ENTER”. All the figures were plotted on the Hiplot website (https://hiplot-academic.com/). All the statistical analyses were performed with the SPSS version 26.0. Statistical significance was defined as a two-sided *p*-value of less than 0.05, and variables were recorded as mean ± SD.

## Results

### The differences in digit ratios between non-PCOS women and men

The baseline information of the study participants is shown in **Table 1**. There was no difference for age and BMI between non-PCOS women and men. The levels of lutein hormone (LH), follicle-stimulating hormone (FSH), and total testosterone (TT) of men were higher than those of non-PCOS women, and the estradiol (E_2_) concentration was lower in men.

**Table 1:** Clinical features of non-PCOS women, PCOS women, and men.

	Non-PCOS *N* = 34	PCOS *N* = 116	Men *N* = 40	*p*-value
Age (years)	29.79 ± 0.81	27.47 ± 4.266^a^	29.67 ± 4.41^b^	0.003
BMI (kg/m^2^)	22.73 ± 0.56	24.94 ± 4.028^a^	24.19 ± 4.71	0.016
LH (IU/L)	4.01 ± 2.70	9.90 ± 6.069^a^	7.78 ± 5.90^ab^	<0.001
FSH (IU/L)	6.25 ± 2.26	5.82 ± 1.612	14.77 ± 13.51^ab^	<0.001
E_2_ (pg/ml)	55.04 ± 46.70	54.93 ± 37.438	36.05 ± 18.86^ab^	0.011
TT (ng/dl)	34.93 ± 9.41	65.85 ± 33.949^a^	396.15 ± 215.16^ab^	<0.001
FNPO	5.25 ± 2.09	12.41 ± 2.95	-	<0.001

ANOVA and Kruskal–Wallis were used to compare the variables among three groups. LSD was used to make pairwise comparison. ^a^, significant difference between non-PCOS and PCOS women; ^b^, significant difference between PCOS women and men.

LH, lutein hormone; FSH, follicle-stimulating hormone; E_2_, estradiol; TT, total testosterone; FNPO, follicle number per ovary.

**Figure 1:**
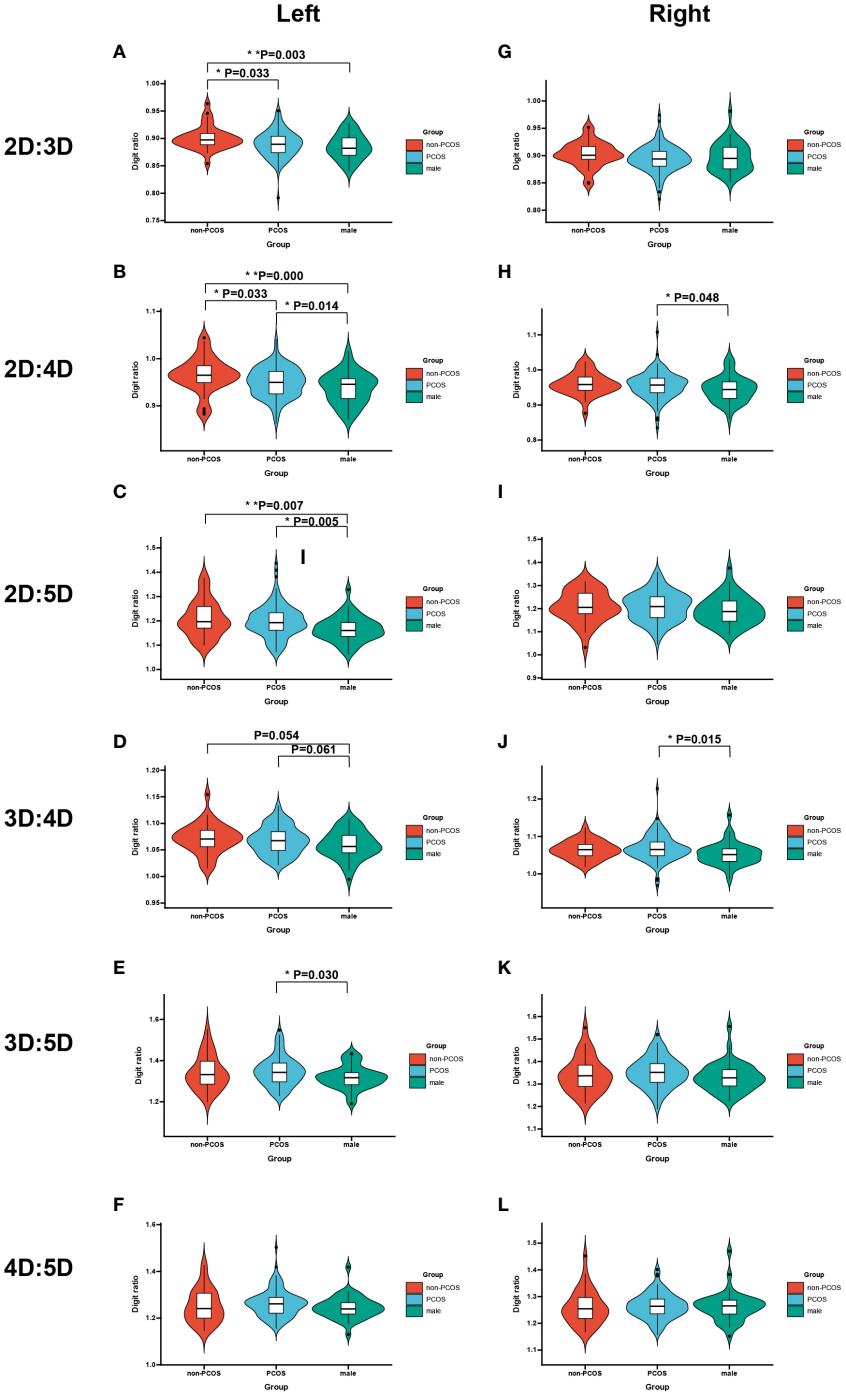
Violin plot shows the mean digit ratio of non-PCOS women, PCOS women, and men (adjusted by age). **(A–F)** The different digit ratios in left hands. **(G–L)** The different digit ratios in right hands. *p*-values were calculated by LSD. * means *p*<0.05 and ** means *p*<0.01.

The average of digit ratios is shown in **Table 2** and **Figure 1**. In general, these digit ratios are listed in order of size: 3D:5D>4D:5D>2D:5D>3D:4D>2D:4D>2D:3D. In the left hands, 2D:3D (*p* = 0.003), 2D:4D (*p*<0.001), and 2D:5D (*p* = 0.007) of non-PCOS women are higher than those of men, indicating the sexual dimorphism of the digit ratios. The other digit ratios showed no difference between men and women.

**Table 2:** The means of 12 digit ratios.

	Non-PCOS	PCOS	Men	*p*-value^*^	*p* trend^#^
L2D:3D	0.9009 ± 0.0214	0.8898 ± 0.0244	0.8850 ± 0.0229	0.011	0.003
L2D:4D	0.9658 ± 0.0370	0.9501 ± 0.0316	0.9377 ± 0.0336	0.001	<0.001
L2D:5D	1.2087 ± 0.0736	1.1991 ± 0.0669	1.1671 ± 0.0524	0.008	0.007
L3D:4D	1.0718 ± 0.0281	1.0679 ± 0.0261	1.0596 ± 0.0262	0.100	0.054
L3D:5D	1.3421 ± 0.0826	1.3480 ± 0.0711	1.3191 ± 0.0559	0.093	0.178
L4D:5D	1.2523 ± 0.0721	1.2622 ± 0.0570	1.2451 ± 0.0486	0.324	0.621
R2D:3D	0.9024 ± 0.0231	0.8942 ± 0.0243	0.8970 ± 0.0266	0.453	0.292
R2D:4D	0.9602 ± 0.0316	0.9553 ± 0.0377	0.9449 ± 0.0366	0.098	0.066
R2D:5D	1.2093 ± 0.0671	1.2082 ± 0.0635	1.1924 ± 0.0626	0.256	0.239
R3D:4D	1.0642 ± 0.0242	1.0684 ± 0.0345	1.0536 ± 0.0306	0.051	0.175
R3D:5D	1.3406 ± 0.0780	1.3514 ± 0.0668	1.3297 ± 0.0634	0.232	0.493
R4D:5D	1.2596 ± 0.0638	1.2650 ± 0.0477	1.2623 ± 0.0548	0.896	0.853

^*^, values of p for differences among three groups (ANOVA); ^#^, values of p for linear trend.

### The differences in digit ratios among non-PCOS and PCOS women

The baseline information of the study participants is shown in **Table 1**. The age of PCOS women was lower than that of non-PCOS women (27.47 ± 4.27 in PCOS women, 29.79 ± 0.81 in non-PCOS women). The average BMI of the PCOS group (24.94 ± 4.03) was higher than that of the non-PCOS group (22.73 ± 0.56). In addition, the endocrine parameters LH and TT concentration and follicle number per ovary of PCOS women were significantly higher than those of non-PCOS women, which were consistent with the endocrine characteristics of PCOS.

Given that PCOS was a female masculinization disease, we compared the digit ratios between non-PCOS and PCOS women. The average of digit ratios is revealed in **Table 2** and **Figure 1**. Generally, left 2D:3D (*p* = 0.003), 2D:4D (*p*<0.001), and 2D:5D (*p* = 0.007) presented a significantly reduced trend among non-PCOS women, PCOS women, and men (**Table 2**). The left 2D:3D (*p* = 0.033) and 2D:4D (*p* = 0.043) ratios of PCOS women were significantly lower than those of non-PCOS women. As for the right hands, no significant difference was found.

### The differences in digit ratios in hyperandrogenic subtypes of PCOS

To further investigate whether the difference in digit ratio was caused by the androgen level, we then divided PCOS women into an HA and an NHA group (**Table 3**). Women with NHA PCOS had an older age than those with HA-PCOS (*p* = 0.043), and BMI is similar between NHA and HA PCOS. The hormonal level (LH, E_2_, and TT) in NHA-PCOS is lower than that in HA-PCOS.

**Table 3:** Clinical features of non-PCOS women, NHA-PCOS women, HA-PCOS women, and men.

	Non-PCOS *N* = 34	NHA-PCOS *N* = 24	HA-PCOS *N* = 82	Men *N* = 40	*p*-value
Age (years)	29.79 ± 0.81	29.21 ± 1.04	27.12 ± 0.45^ab^	29.67 ± 4.41^c^	0.003
BMI (kg/m^2^)	22.73 ± 0.56	24.77 ± 0.66	25.25 ± 0.48^a^	24.19 ± 4.71	0.022
LH (IU/L)	4.01 ± 2.70	5.22 ± 3.05	11.25 ± 6.17^ab^	7.78 ± 5.90^ac^	<0.001
FSH (IU/L)	6.25 ± 2.26	5.38 ± 1.57	5.94 ± 1.63	14.77 ± 13.51^abc^	<0.001
E_2_ (pg/ml)	55.04 ± 46.70	39.89 ± 22.43^a^	55.82 ± 27.64^b^	36.05 ± 18.86^ac^	<0.001
TT (ng/dl)	34.93 ± 9.41	32.15 ± 16.95	75.71 ± 31.27^ab^	396.15 ± 215.16^abc^	<0.001

p-value was given by ANOVA and Kruskal–Wallis test to compare differences among groups. LSD was used to make pairwise comparison. ^a^, significant difference compared with the non-PCOS group; ^b^, significant difference compared with the NHA-PCOS group; ^c^, significant difference compared with the HA-PCOS group.

LH, lutein hormone; FSH, follicle-stimulating hormone; E_2_, estradiol; TT, total testosterone.

**Figure 2:**
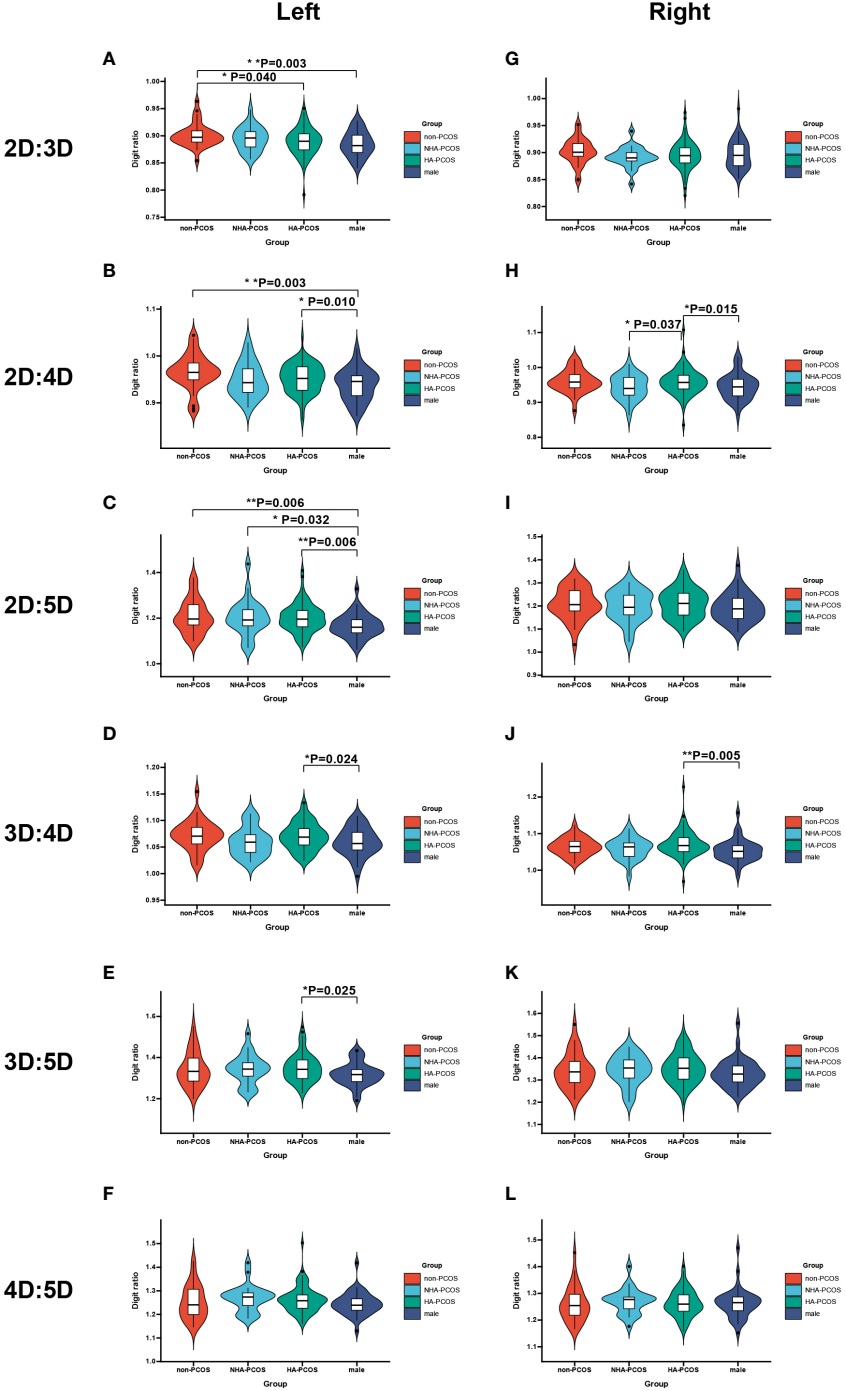
Violin plot shows the mean digit ratio of non-PCOS women, NHA-PCOS women, HA-PCOS, and men (adjusted by age). **(A–F)** The different digit ratios in left hands. **(G–L)** The different digit ratios in right hands. *p*-values were calculated by LSD. * means *p*<0.05 and ** means *p*<0.01.

The average of digit ratio is listed in **Table 4**. Overall, 2D:3D, 2D:4D, and 2D:5D of left hands maintained a downward trend (*p* = 0.002, *p* = 0.001, and *p* = 0.010), in which both 2D:3D and 2D:5D in HA-PCOS were lower than those in NHA-PCOS without statistical significance (**Figure 2**). However, the left 2D:4D ratio in HA-PCOS was higher than that in NHA-PCOS.

**Table 4:** The means of digit ratios in non-PCOS women, NHA-PCOS women, HA-PCOS women, and men.

	Non-PCOS	NHA-PCOS	HA-PCOS	Men	*p*-value^*^	*p* trend^#^
L2D:3D	0.9009 ± 0.0214	0.8947 ± 0.0220	0.8896 ± 0.0255	0.8850 ± 0.0229	0.025	0.002
L2D:4D	0.9658 ± 0.0370	0.9481 ± 0.0358	0.9518 ± 0.0308	0.9377 ± 0.0336	0.003	0.001
L2D:5D	1.2087 ± 0.0736	1.2028 ± 0.0798	1.2007 ± 0.0618	1.1671 ± 0.0524	0.018	0.010
L3D:4D	1.0718 ± 0.0281	1.0596 ± 0.0269	1.0700 ± 0.0255	1.0596 ± 0.0262	0.047	0.211
L3D:5D	1.3421 ± 0.0826	1.3437 ± 0.0717	1.3502 ± 0.0680	1.3191 ± 0.0559	0.163	0.244
L4D:5D	1.2523 ± 0.0721	1.2680 ± 0.0565	1.2617 ± 0.0547	1.2451 ± 0.0486	0.423	0.517
R2D:3D	0.9024 ± 0.0231	0.8916 ± 0.0190	0.8951 ± 0.0259	0.8970 ± 0.0266	0.464	0.495
R2D:4D	0.9602 ± 0.0316	0.9436 ± 0.0343	0.9584 ± 0.0369	0.9449 ± 0.0366	0.031	0.257
R2D:5D	1.2093 ± 0.0671	1.1989 ± 0.0661	1.2102 ± 0.0634	1.1924 ± 0.0626	0.355	0.411
R3D:4D	1.0642 ± 0.0242	1.0583 ± 0.0289	1.0711 ± 0.0342	1.0536 ± 0.0306	0.031	0.444
R3D:5D	1.3406 ± 0.0780	1.3445 ± 0.0661	1.3526 ± 0.0675	1.3297 ± 0.0634	0.433	0.618
R4D:5D	1.2596 ± 0.0638	1.2702 ± 0.0476	1.2631 ± 0.0495	1.2623 ± 0.0548	0.901	0.976

^*^, values of p for differences among three groups (ANOVA); ^#^, values of p for linear trend.

### Predictors of PCOS using regression models of digit ratios

Based on the above findings, a logistic regression model was constructed to investigate the potential of digit ratio as an anatomical marker in PCOS. The results revealed that 2D:3D (*p* = 0.013), 2D:4D (*p* = 0.006), 2D:5D (*p* = 0.025), and 3D:4D (*p* = 0.013) of left hands were statistically significant in the constructed model (**Table 5**), indicating their crucial effect on the development of PCOS and the possibility of them being effective markers for PCOS.

**Table 5:** The results of the logistic regression model.

	*p*-value
L2D:3D	0.013
L2D:4D	0.006
L2D:5D	0.025
L3D:4D	0.013

The independent variables, including 12 digit ratios of right and left hands, were forced simultaneously into the model, and the dependent variable was PCOS.

In summary, our results emphasized the significance of left 2D:3D and left 2D:4D, both of which presented a reduced tendency among different populations, that is, non-PCOS women>PCOS women>men.

## Discussion

In this study, we demonstrated that left 2D:3D, 2D:4D, and 2D:5D showed sexual dimorphism; both 2D:3D and 2D:4D of left hands in PCOS women were statistically lower than those in non-PCOS women, and both 2D:3D and 2D:5D of left hands in HA-PCOS were lower than those in NHA-PCOS without statistical significance by systematical measurement of all the digit ratios. The logistic regression model revealed that 2D:3D, 2D:4D, 2D:5D, and 3D:4D of left hands were statistically related to the diagnosis of PCOS. Our results indicated that, apart from 2D:4D, other digit ratios, such as 2D:3D and 2D:5D, might be an anatomical marker of PCOS. It is worth mentioning that all of the significant digit ratios involved left 2D.

There was a decreased trend of several digit ratios among three groups, in which left digit ratios were significantly distinct in these groups. The decreased trend pointed out that the digit ratios of PCOS women were lower than those of non-PCOS women and higher than those of men, especially for ratios that included left 2D. Firstly, significant sexual dimorphism was also observed in the left 2D:3D, 2D:4D, and 2D:5D. A similar pattern has been observed in previous studies (15, 18), suggesting that sexual dimorphism of digit ratio not only was confined to 2D:4D, but also included 2D:3D and 2D:5D. Secondly, digit ratios (2D:4D of left hands) in PCOS women were lower than those in non-PCOS women, which was consistent with previous studies (11, 14). Moreover, PCOS was reported to be associated with delayed menarche (19, 20), and individuals with delayed menarche had lower 2D:4D (21). Lujan et al. found no difference in 2D:4D between PCOS and controls, which was the opposite of our conclusion (12, 13). The inconsistency might result from the large difference in BMI between the two groups, and they analyzed the digit ratio without adjusting BMI. In our study, there was no significant link between digit ratio and BMI. Consequently, our data analysis was not adjusted by BMI. In addition, the statistical significance of 2D:4D remained even after adjusting BMI. The second reason for these inconsistent results can be explained by ethnicity difference in 2D:4D, which has been reported in a number of studies (5, 22, 23). Thirdly, there was a higher difference in left hands than in right hands, which was opposite from the typical conclusion that right 2D:4D is the significantly different digit ratio between men and women. The inconsistent results is common in a Chinese population; for example, the left-hand 2D:4D tended to be closely associated with the corresponding disease in the study of schizophrenia (24), neuroticism (25), and breast cancer (26). It has been reported that the prevalence of left-handedness was lower in Asia due to biological or cultural factors (27, 28). Besides that, previous studies confirmed that left-handedness was linked to low R2D:4D and high L2D:4D in the same individual (29). Richards et al. speculated that gripping pens and pencils in writing led to the association between handedness and digit ratio (30). We hypothesized that the higher significance in left hands might result from the change for the size of right hands due to the long-time use in China: the right hand of right-handedness has been reported to be larger than the left (31, 32).

To further investigate the prenatal androgen effect on the digit ratio in PCOS cases, we divided PCOS into an HA and an NHA subgroup. We hypothesized that the severe subtype of PCOS (HA-PCOS) tends to expose more fetal T and has a masculinized digit ratio. Consistent with the hypothesis, the digit ratio of left 2D:3D and 2D:5D presented a decreased tendency from non-PCOS women to NHA-PCOS women to HA-PCOS women. However, the left 2D:4D ratio in the HA-PCOS subgroup was higher than that in the NHA-PCOS subgroup, which may result from the higher estradiol concentration in HA-PCOS. Manning et al. reported that the 2D:4D ratio in women was positively related with estradiol (1), and the amnionic study illustrated that the 2D:4D ratio was positively associated with the ratio of fetal testosterone and fetal estradiol (3), suggesting that the estradiol concentration had an influence on the 2D:4D ratio. Therefore, 2D:4D might not be a good marker for identifying NHA and HA-PCOS, whereas left 2D:3D and 2D:5D may or may not be an anatomical marker for hyperandrogenism, which needs further investigation using large cohort studies.

Based on the above results, a logistic regression model with all the digit ratios forced simultaneously into the model was constructed to explore the significance of digit ratio in PCOS identification. Strikingly, the left 2D:3D, 2D:4D, 2D:5D, and 3D:4D ratios were significant in the model, which re-emphasized that left digit ratios were better than right digit ratios and that not only 2D:4D but also 2D:3D, 2D:5D, and 3D:4D represented the prenatal androgen exposure. Moreover, the 2D:3D, 2D:4D, and 2D:5D ratios shared the index finger, indicating that the second digit length tended to be shorter with more androgen exposure. 2D:4D and 3D:4D shared the ring finger, suggesting that the fourth digit length tended to be longer with testosterone exposure. Similarly, the nonhuman study of rats and rhesus monkeys exposed to testosterone in early-to-mid gestation presented masculinized 2D length and a PCOS-like phenotype (33) (34). Zheng and Cohn (35) and Manning (36) have reported the potential mechanism that both Androgen receptor (AR) and estrogen receptor involved the development of finger length in mice and androgen excess acted on AR in 4D to increase the length.

The major strength of this study was the systematical measurement of all digit ratios and the fact that it presented an intact pattern of all digit ratios in non-PCOS women, PCOS women, and men, providing more striking evidence for the digit ratio as a marker of prenatal testosterone. We also acknowledged that there were some limitations in our study. We hypothesized that left-hand 2D:4D might be a more effective marker for a Chinese PCOS population without direct evidence for validation.In conclusion, we revealed that left digit ratios might be a better marker of prenatal androgen exposure than right digit ratios in China and provided insight into the anatomical evidence of prenatal androgen exposure in PCOS. In addition to 2D:4D, significant sexual dimorphism was observed in 2D:3D and 2D:5D. 2D:3D, 2D:4D, 2D:5D, and 3D:4D were statistically significant in the logistic model of PCOS, and the first three items were masculinized in PCOS; that is, these digit ratios were lower than non-PCOS. More detailed analysis revealed no significant difference between the NHA and HA PCOS subgroups. These results suggested the significance of the digit left 2D. We found the magnitude of the left 2D:3D and 2D:4D ratios to be non-PCOS women>PCOS women>men.

## Data availability statement

The raw data supporting the conclusions of this article will be made available by the authors, without undue reservation.

## Ethics statement

The studies involving human participants were reviewed and approved by KYLL-2021-1070. The patients/participants provided their written informed consent to participate in this study.

## Author contributions

All authors contributed to the study conception and design. Study design: XY. Photocopies collection: AZ and YL. Point marker on the photocopies: ZY and YW. Digit-length measurement: LL and WL. Digit-length remeasurement: DL and FL. Formal analysis: JD and FC. Data analysis: XY. Writing—original draft preparation: XY. Writing—review and editing: XY and XG. Supervision: XG and JZ. All authors contributed to the article and approved the submitted version.
